# Proteomic analysis of the umbilical cord in fetal growth restriction and preeclampsia

**DOI:** 10.1371/journal.pone.0262041

**Published:** 2022-02-25

**Authors:** Matthew S. Conrad, Miranda L. Gardner, Christine Miguel, Michael A. Freitas, Kara M. Rood, Marwan Ma’ayeh

**Affiliations:** 1 Department of Obstetrics and Gynecology, The Ohio State University College of Medicine, Columbus, Ohio, United States of America; 2 Campus Chemical Instrument Center (CCIC), Mass Spectrometry and Proteomics Facility, The Ohio State University College of Medicine, Columbus, Ohio, United States of America; Rutgers University/New Brunswick, UNITED STATES

## Abstract

Fetal growth restriction (FGR) is associated with adverse perinatal outcomes. Pre-eclampsia (PreE) increases the associated perinatal morbidity and mortality. The structure of the umbilical cord in the setting of FGR and PreE is understudied. This study aimed to examine changes in the umbilical cord (UC) composition in pregnancies complicated by FGR and FGR with PreE. UC from gestational age-matched pregnancies with isolated FGR (n = 5), FGR+PreE (n = 5) and controls (n = 5) were collected, and a portion of the UC was processed for histologic and proteomic analysis. Manual segmentation analysis was performed to measure cross-section analysis of umbilical cord regions. Wharton’s Jelly samples were analyzed on a tims-TOF Pro. Spectral count and ion abundance data were analyzed, creating an intersection dataset from multiple mass spectrometry search and inference engines. UCs from FGR and FGR with PreE had lower cross-sectional area and Wharton’s Jelly area compared with control (p = 0.03). When comparing FGR to control, 28 proteins were significantly different in abundance analysis and 34 in spectral count analysis (p < 0.05). Differential expression analysis between PreE with FGR vs controls demonstrated that 48 proteins were significantly different in abundance and 5 in spectral count. The majority of changes occurred in proteins associated with extracellular matrix, cellular process, inflammatory, and angiogenesis pathways. The structure and composition of the UC is altered in pregnancies with FGR and FGR with PreE. Future work in validating these proteomic differences will enable identification of therapeutic targets for FGR and FGR with PreE.

## Introduction

Fetal growth restriction (FGR) is a common condition that leads to a variety of adverse perinatal and postnatal outcomes. FGR is defined as an estimated fetal weight of less than the 10^th^ percentile [1]. FGR increases the risk of neonatal morbidity and mortality including stillbirth as well as long term morbidity such as cognitive delay, obesity and coronary heart disease [2, 3]. FGR can be the result of maternal, fetal, and/or placental mechanisms. Of these, the most common mechanism is placental or umbilical cord disorders [4].

Pre-eclampsia (PreE) is also a major risk factor for development of FGR. PreE affects 3–8% of all pregnancies and leads to increased morbidity and mortality for both the mother and the fetus [5]. In addition to increased risk of FGR, PreE increases risk of preterm delivery, placental abruption, neonatal respiratory distress syndrome, cerebral palsy and perinatal death [5]. The etiology of PreE is multifactorial, and the pathophysiology of its fetal growth restrictive effects is not fully understood.

The umbilical cord connects the developing fetus to the placenta. The umbilical cord is composed of two umbilical arteries, one umbilical vein, and an extracellular matrix (ECM) surrounding these structures called Wharton’s Jelly. It is thought that changes in the umbilical cord, including fibrosis, may lead to stricture or hypercoiling of the umbilical cord, obstructing uteroplacental blood flow [6]. Research is limited, however, on morphology of the umbilical cord, how the composition of Wharton’s Jelly changes in FGR and PreE, and its role in these disease processes.

Wharton’s Jelly is highly abundant in collagen and glycosaminoglycan (GAG) proteins [7]. In PreE, there is a shift to an increase in sulphated GAGs from hyaluronic acid. This shift in equilibrium may represent a “premature aging” of the tissue with contribution to the development of FGR [8]. However, little is known about the changes in the composition of Wharton’s Jelly in FGR fetuses and warrants further investigation.

Umbilical cords from FGR pregnancies have demonstrated changes in the umbilical cord morphology including decreased umbilical cord diameters, cross-sectional area and decreased umbilical artery and vein area [9, 10]. These findings were further confirmed with ultrasound morphometry measurements [11]. Interestingly, the cross-sectional areas of the vessels are larger in the umbilical cords of babies born to women with PreE [12]. Yet, the volume of the whole umbilical cord and changes to the area of Wharton’s Jelly in PreE has not been studied. These changes in morphology may contribute to the development of FGR due to changes in fetal blood flow.

Mass spectrometry-based proteomics has emerged as a promising high-throughput technology for identification of potential biomarker candidates for diseases. Recent reviews have highlighted the proteomic approaches that have been used to explore PreE, FGR and preterm birth [13–15]. This data has been complicated by a wide variety of techniques used and tissues analyzed. Despite this variability, meta-analysis has identified common proteins across multiple studies that are differentially expressed [14]. None of these studies, however, have looked at the Wharton’s Jelly and the differential protein expression in FGR and PreE.

The study’s primary hypothesis is that the umbilical cord structure and proteomic composition of Wharton’s Jelly is altered in women with FGR and PreE + FGR. Specifically, these changes will include decreased cross-sectional area of the umbilical cord and proteomic changes in extracellular matrix proteins.

## Methods

### Screening and enrollment

Patients who presented to labor and delivery with singleton gestations were considered for this study. The study design was a case-control study with groups as follows: FGR, FGR with PreE, and gestational age-matched controls (n = 5 for each group). Gestational age was determined by last menstrual period and ultrasound biometry before 20 weeks using standard criteria [16]. Patients were consented for the study by trained research personnel and clinical care was at discretion of their provider. Demographic data was collected through a combination of interview with the patient and the electronic medical record. Delivery and neonatal outcomes were assessed through the electronic medical record. Data collected included gestational age, maternal age, parity, delivery route, Body Mass Index, chronic hypertension, diabetes, neonatal APGAR scores, neonatal sex, and neonatal weight. Subjects were recruited, and written informed consent obtained, under the Ohio State University Institutional Review Board (Study Number: 2019H0039).

### Sample collection

The umbilical cords were collected after delivery and placed under refrigeration within one h. A standardized 1cm segment was collected approximately 5cm from the cord placental insertion site and placed in 10% formalin for histology within 6 h from delivery. Wharton’s Jelly was dissected from the umbilical cord, immediately frozen at -80˚C and stored until further processing.

### Umbilical cord histological analysis

The 1cm segments of umbilical cord were previously placed in 10% formalin. The samples were processed by the Comparative Pathology and Mouse Phenotyping Shared Resource. They were embedded in paraffin, sectioned at 4μm, and then stained with hematoxylin and eosin. They were then digitally scanned by using an Aperio Digital Pathology System (Leica, Illinois, USA). ImageJ was used for area analysis of the umbilical cord using manual segmentation. Cross sectional area measurements were computed for umbilical cord area, Wharton’s Jelly area, average artery tunica media outer, average artery tunica media inner, and vein wall area.

### Statistical analysis

Statistical analysis was performed using SPSS (IBM, Armonk, NY, USA). Baseline patient characteristics were compared using one-way ANOVA with Bonferroni post-hoc testing. The APGAR data is presented as medians with interquartile range with comparison using a Kruskal-Wallis test. Umbilical cord region of interest area means were compared with a general linear model with Bonferroni post-hoc testing with correction for gestational age. Significance threshold was considered at p-value < 0.05.

### Sample preparation for proteomics

At time of collection, a portion of Wharton’s Jelly was dissected and frozen at -80˚C. Samples were washed twice with 50 mM ammonium bicarbonate. Approximately 43 mg of Biorupter sonication beads were then added to the sample, along with 100 μL of 50 mM ammonium bicarbonate containing 0.1% Rapigest (Waters Corp). Samples were sonicated in a Biorupter (Diagenode) with 20 on/off cycles using 30 sec on and 30 sec off. Extracts were spun at 13K rpm in a microcentrifuge to pellet debris and supernatant was transferred to a new tube. The supernatant was incubated with DTT (5 mM final concentration) at 65°C for 30 minutes. The supernatant was then incubated with iodoacetamide (15 mM final concentration) in the dark at room temperature for 30 minutes. Trypsin (1 ug, sequencing grade, Promega) was added for digestion, and samples were then incubated at 37°C overnight. The following day, digestion was quenched with addition of trifluoroacetic acid (final concentration 0.5%) and sample was incubated at 37°C for 30 minutes to precipitate the Rapigest. The sample was clarified at 13K rpm for 5 min in a microcentrifuge, supernatant dried down in a vacufuge (Eppendorf), and desalted with a Ziptip. After desalting, samples were dried down in a vacufuge prior to resuspension in water with 0.1% formic acid and determination of peptide concentration via nanodrop (A280nm).

### LC-MS/MS

Protein identification was performed on the supernatant from the protein digestion of the Wharton Jelly samples using nano-liquid chromatography-nanospray tandem mass spectrometry (LC/MS/MS) on a Bruker tims-ToF Pro equipped with a CaptiveSpray source operated in positive ion mode. Samples (200ng injection) were separated on a C_18_ reverse phase column (1.6 μm, 250mm* 75 μm IonOpticks) using a Bruker nanoElute UHPLC system. Pre-injection column equilibration consisted of 4 column volumes at 800 bar. Mobile phase A was 0.1% Formic Acid in water while acetonitrile (with 0.1% formic acid) was used as mobile phase B. A flow rate of 0.4 μL/min was used. Mobile phase B was increased from 2 to 17% over the first 60 min, increased to 25% over the next 10 min, further increased to 37% over the next 10 min, and finally increased to 80% over 10 min and then held at 80% for 10 min.

MS and MS/MS experiments were recorded over the *m/z* range 100–1700 and K_0_ of 0.6–1.6. PASEF was used for all experiments, with the number of PASEF MS/MS scans set to 10. Active exclusion was applied, releasing after 0.4 min, with precursor reconsidered if current intensity/previous intensity was 4.0 or greater.

### Protein identification, data analysis and statistics for proteomics

For this study, a bottom-up shotgun quantitative proteomic approach was considered for identification of differentially expressed proteins in Wharton’s Jelly. A combination of label-free quantification strategies, peak intensity and spectral count [17, 18], were used to analyze the data. Briefly, raw.d files generated from the timsTOF Pro were converted to mzML with OpenMS (v 2.5.0) and tdf2mzml in-house nextflow script. Converted mzML files were searched against a reviewed UniProt human proteome (downloaded 1/1/2020) in OpenMS with the following protein search engine and inference engine combination: Comet fido, Comet epiphany, X!Tandem epiphany, MSGF+ fido, and MSGF+ epiphany [19–22]. Search parameters included precursor mass tolerance 20 ppm, MS2 mass tolerance 0.05 Da, carbamidomethylation of cysteine as a fixed modification, oxidation of methionine as a variable modification and false discovery rate (FDR) of peptide and proteins equal to 0.05.

For differential expression analysis with spectral count data, samples were trimmed mean normalized, and significance (p-value < 0.05) determined by edgeR generalized linear model quasi-likelihood Ftest (glmQLFTest) as described in [23]. Resulting p-values were adjusted for multiple hypothesis testing with Bonferroni-Hochberg method. For differential expression analysis with peak intensity data, missing values were imputed according to sample group, described by Gardner *et al*. [24]. Data was quantile normalized, and significance (p-value < 0.05) determined by a modified exact test. Downstream gene ontology, KEGG and pathway analyses utilized the list of significant proteins identified across the database combination for each pair-wise comparison, reporting the log fold-change values as well. Protoemics data has been deposited to ProteomeXchange through massIVE and can be accessed through the dataset identifier PXD024751 or ftp link (ftp://massive.ucsd.edu/MSV000087051/).

## Results

### Histology

Characteristics of the study population are shown in Table 1. There was no significant difference found in the maternal characteristics including maternal age, BMI, presence of chronic hypertension, or maternal diabetes (pre-gestational or gestational). There was no significant difference in fetal gestational age at time of delivery (p = 0.36). There was a significant difference in route of delivery where all the controls were vaginal deliveries and the FGR and FGR with PreE were majority cesarean delivery (p < 0.01). The indication for cesarean delivery was non-reassuring fetal testing in all cases. The indication for delivery for the controls were spontaneous preterm labor or PPROM. There were no significant differences in 1 min and 5 min APGAR scores, parity, or neonatal sex between groups. There was a trend for difference in neonatal weight with FGR and FGR with PreE less than controls (p = 0.09).

**Table 1:** Demographics and clinic assessment of study population.

	Mean (SD) Control	FGR	FGR + PreE	P-Value
Number	5	5	5	-
Gestational Age (wks)	35.1 (3.3)	35.3 (3.1)	32.9 (1.4)	0.362
Maternal Age (years)	26.2 (4.8)	31.6 (5.0)	29 (10.5)	0.520
Parity	1.8 (0.4)	1.4 (0.5)	1.2 (0.4)	0.397
Delivery Route - Vaginal	5	1	1	**0.006**
-Cesarean	0	4	4	
BMI	27.5 (6.5)	36.2 (9.2)	40.2 (12.9)	0.211
cHTN	0	1	2	0.335
Diabetes	1	2	2	0.422
Neonate Sex - Male	4	4	1	0.088
-Female	1	1	4	
Neonate Weight (g)	2361.2 (550.6)	1944.20 (810.8)	1431.6 (351.40)	0.088
	Median (25%ile,75%ile)			
AGARS 1 min	8 (7,8)	8 (6,9)	8 (2,8)	0.781
APGARS 5 min	9 (9,9)	9 (8,9)	9 (9,9)	>0.999

**Fig 1:**
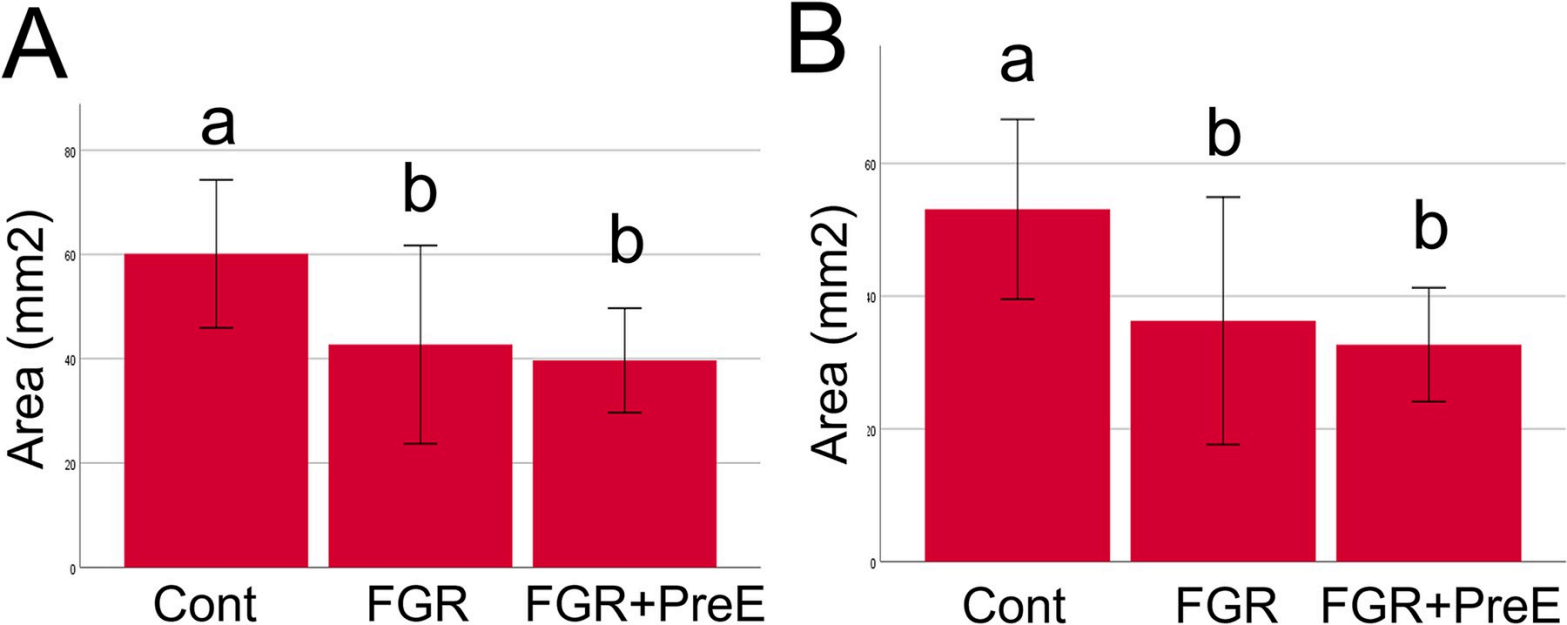
Umbilical cord total area and Wharton’s Jelly area. Umbilical cord total area with groups control, FGR, FGR + PreE (A, p = 0.03). Wharton’s Jelly area (B, p = 0.03). Fetal Growth Restriction (FGR), Preeclampsia (PreE). Bonferroni post-hoc analysis with significantly different groups indicated by letter above bar.

Histological analysis of the umbilical cord showed that the cross-sectional total umbilical cord area is significantly different between the groups with the control groups having a significantly larger area compared to the FGR and FGR+PreE groups (Fig 1, p = 0.03) when corrected for gestational age. Similarly, there is a significant difference in the Wharton’s Jelly area with the control groups having a significantly larger area compared to the FGR and FGR+PreE groups (Fig 1, p = 0.03). There were no significant differences found in the artery average tunica media outer area (S1 Fig). The average of the FGR with PreE arterial tunica media inner layer was larger than controls and FGR, but this did not reach significance (S2 Fig, p = 0.75). There were no differences in the umbilical cord vein wall area (S3 Fig).

### Proteomics

**Fig 2:**
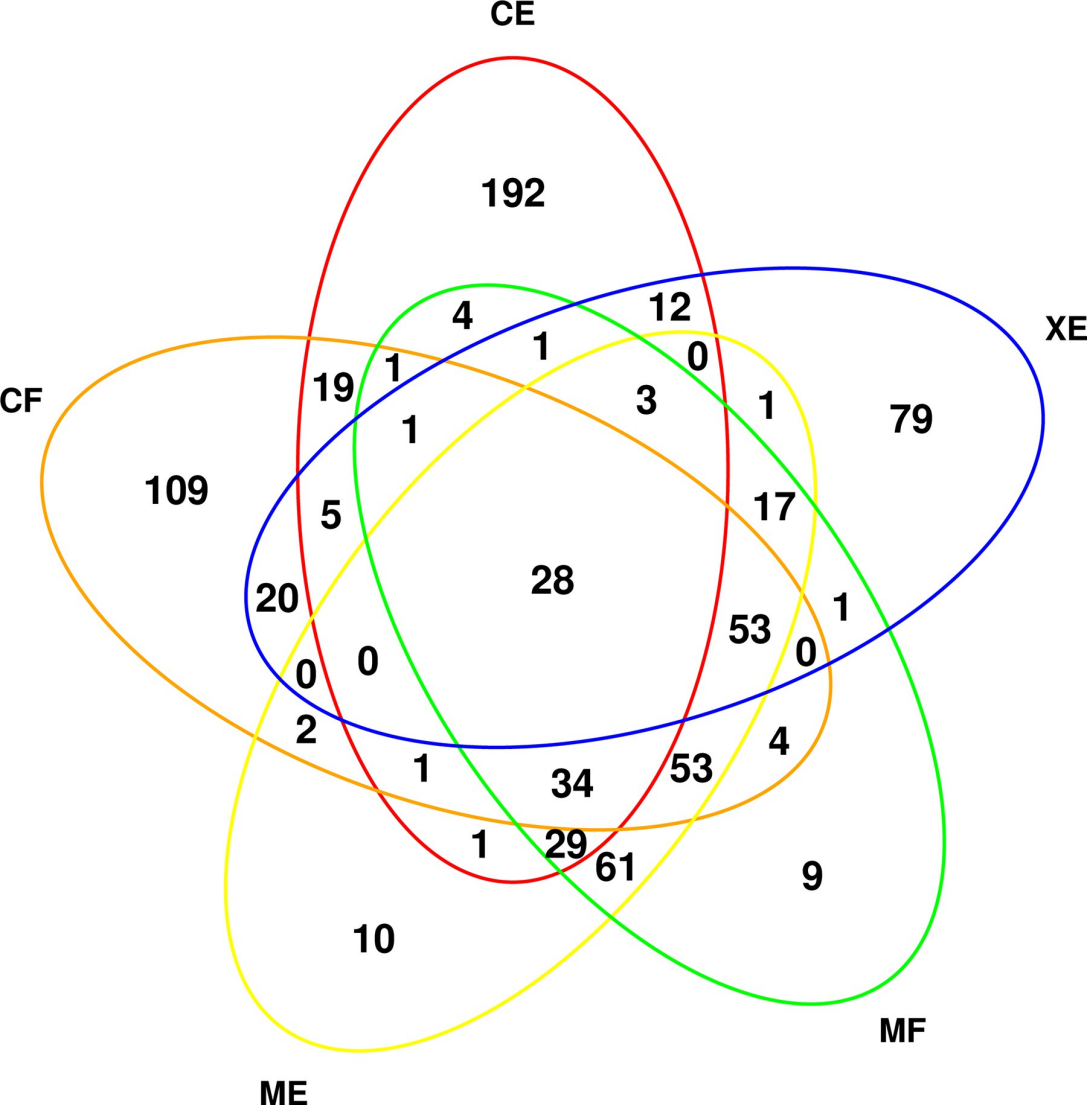
Overlap of significantly changed protein ID’s from mass spectrometry search and inference engines for peak intensity of FGR versus controls. Legend (CF: comet fido; CE: comet epifany; XE: X!Tandem epifany; MF: MSGF fido; ME: MSGF epiphany).

A novel comparison technique for identification of proteins was used in this study. Five combinations of protein identification databases (Comet fido, Comet epiphany, X!Tandem epiphany, MSGF+ fido, and MSGF+ epiphany) were used to generate a common overlap dataset containing over 1000 proteins present in each pair-wise comparison. As seen in Fig 2, peak intensity analysis for FGR versus controls resulted in 28 common differentially expressed proteins across the five different database combinations. Fourteen proteins were downregulated and fourteen upregulated with the largest log fold-change (logFC) values include Prolyl 3-hydroxylase 3, Immunoglobulin heavy constant gamma 4, Procollagen C-endopeptidase enhancer 1, COP9 signalosome complex subunit 3 and Cyclin-dependent kinase 6 (S1 Table). Thirty-four proteins were significantly changed for FGR versus controls using spectral count. Twenty-three were downregulated and eleven upregulated with largest logFC included Matrix-remodeling-associated protein 5, Pregnancy zone protein, Signal transducer and activator of transcription 6, and Filamin-binding LIM protein 1 (S4 Fig, Table 2). Forty-eight proteins were significantly changed for FGR with PreE versus controls using peak intensity (S5 Fig) with twenty being downregulated and twenty-eight being upregulated. The proteins with the largest logFC included Eukaryotic translation initiation factor 3 subunit E, Protein enabled homolog, Desmocollin-2and Tripeptidyl-peptidase 2 (Table 3). Five proteins were significantly changed for FGR with PreE versus controls using spectral count with two being downregulated and three being upregulated (S6 Fig, S2 Table). Eighty proteins were significantly changed for FGR with PreE versus FGR using peak intensity with twenty-eight being downregulated and fifty-two being upregulated. The largest changes were seen in Sideroflexin-3, Mannose-1-phosphate guanyltransferase alpha, Lectin, galactoside-binding, soluble, 7B and Desmoglein-3 (S7 Fig, Table 4). Zero proteins were significantly different for FGR with PreE versus FGR using spectral count analysis (S8 Fig).

**Table 2:** Intersection of significantly changed protein expression for FGR versus controls spectral count.

UniProt ID	Protein	Log Fold Change
Q9NR99	Matrix-remodeling-associated protein 5	-3.32
P20742	Pregnancy zone protein	-3.13
O75339	Cartilage intermediate layer protein 1	-2.90
Q32P28	Prolyl 3-hydroxylase 1	-2.61
P12107	Collagen alpha-1(XI) chain	-2.49
P25940	Collagen alpha-3(V) chain	-2.39
P49746	Thrombospondin-3	-2.16
Q12884	Prolyl endopeptidase FAP	-2.14
P62263	40S ribosomal protein S14	-2.06
P78539	Sushi repeat-containing protein SRPX	-2.00
A1L4H1	Soluble scavenger receptor	-1.88
Q13421	Mesothelin	-1.85
P55287	Cadherin-11	-1.57
Q14257	Reticulocalbin-2	-1.36
P01861	Immunoglobulin heavy constant gamma 4	-1.27
Q07092	Collagen alpha-1(XVI) chain	-1.17
P02771	Alpha-fetoprotein	-0.98
Q02809	Procollagen-lysine,2-oxoglutarate 5-dioxygenase 1	-0.96
Q9NRN5	Olfactomedin-like protein 3	-0.93
Q96CN7	Isochorismatase domain-containing protein 1	-0.91
Q9HCB6	Spondin-1	-0.74
O00339	Matrilin-2	-0.50
P02452	Collagen alpha-1(I) chain	-0.43
Q14112	Nidogen-2	1.06
P00390	Glutathione reductase, mitochondrial	1.18
P126112	Aggrecan core protein	1.26
P31040	Succinate dehydrogenase	1.31
Q7Z4W1	L-xylulose reductase	1.72
O14672	Disintegrin and metalloproteinase domain-containing protein 10	2.09
P48960	Adhesion G protein-coupled receptor E5	2.16
Q9HAV0	Guanine nucleotide-binding protein subunit beta-4	2.38
P36269	Glutathione hydrolase 5 proenzyme	2.39
Q8WUP2	Filamin-binding LIM protein 1	2.43
P42226	Signal transducer and activator of transcription 6	3.00

**Table 3:** Intersection of significantly changed protein expression for FGR+PreE versus controls peak intensity.

UniProt ID	Protein	Log Fold Change
Q8N8S7	Protein enabled homolog	-4.07
P60228	Eukaryotic translation initiation factor 3 subunit E	-2.65
P62191	26S proteasome regulatory subunit 4	-2.54
Q13576	Ras GTPase-activating-like protein IQGAP2	-2.26
Q16881	Thioredoxin reductase 1, cytoplasmic	-2.09
Q02818	Nucleobindin-1	-1.93
O95373	Importin-7	-1.90
P23381	Tryptophan—tRNA ligase, cytoplasmic	-1.90
P56134	ATP synthase subunit f, mitochondrial	-1.88
Q9BTT0	Acidic leucine-rich nuclear phosphoprotein 32 family member E	-1.84
Q9Y446	Plakophilin-3	-1.82
Q13361	Microfibrillar-associated protein 5	-1.77
P30711	Glutathione S-transferase theta-1	-1.58
P04181	Ornithine aminotransferase, mitochondrial	-1.49
P01721	Immunoglobulin lambda variable 6–57	-1.45
P09110	3-ketoacyl-CoA thiolase, peroxisomal	-1.37
P06276	Cholinesterase	-1.36
P13693	Translationally-controlled tumor protein	-1.34
P55001	Microfibrillar-associated protein 2	-1.26
Q9Y3Z3	Deoxynucleoside triphosphate triphosphohydrolase SAMHD1	-1.12
Q8NB37	Glutamine amidotransferase-like class 1 domain	1.09
P27169	Serum paraoxonase/arylesterase 1	1.13
Q14192	Four and a half LIM domains protein 2	1.39
Q13325	Interferon-induced protein with tetratricopeptide repeats 5	1.47
Q96CX2	BTB/POZ domain-containing protein KCTD12	1.47
P35268	60S ribosomal protein L22	1.49
P01834	Immunoglobulin kappa constant	1.49
Q6XQN6	Nicotinate phosphoribosyltransferase	1.67
O75489	NADH dehydrogenase [ubiquinone] iron-sulfur protein 3	1.79
P20810	Calpastatin	1.84
P52566	Rho GDP-dissociation inhibitor 2	1.88
Q8TDZ2	[F-actin]-monooxygenase MICAL1	1.94
P01236	Prolactin	2.00
P19827	Inter-alpha-trypsin inhibitor heavy chain H1	2.04
P12956	X-ray repair cross-complementing protein 6	2.04
P20700	Lamin-B1	2.12
P06702	Protein S100-A9	2.22
P07476	Involucrin	2.28
P02746	Complement C1q subcomponent subunit B	2.38
Q9NUQ9	CYFIP-related Rac1 interactor B	2.42
P53634	Dipeptidyl peptidase 1	2.48
P46459	Vesicle-fusing ATPase	2.58
P29992	Guanine nucleotide-binding protein subunit alpha-11	2.73
Q9UBC9	Small proline-rich protein 3	2.74
P47929	Galectin-7	3.13
P16144	Integrin beta-4	3.20
Q02487	Desmocollin-2	3.22
P29144	Tripeptidyl-peptidase 2	3.42

**Table 4:** Intersection of significantly changed protein expression for FGR+PreE versus FGR peak intensity.

UniProt ID	Protein	Log Fold Change
Q9BWM7	Sideroflexin-3	-5.08
Q96IJ6	Mannose-1-phosphate guanyltransferase alpha;	-2.97
P35998	26S proteasome regulatory subunit 7	-2.97
P08574	Cytochrome c1, heme protein, mitochondrial	-2.87
Q9UNS2	COP9 signalosome complex subunit 3	-2.69
P30837	Aldehyde dehydrogenase X, mitochondrial	-2.66
P00367	Glutamate dehydrogenase 1	-2.54
P07357	Complement component C8 alpha chain	-2.41
O15061	Synemin; Type-VI intermediate filament	-2.25
Q15717	ELAV-like protein 1	-2.19
P35573	Glycogen debranching enzyme	-2.17
Q00534	Cyclin-dependent kinase 6	-2.15
Q2M389	WASH complex subunit 4	-2.10
O15230	Laminin subunit alpha-5;	-2.05
P54136	Arginine—tRNA ligase, cytoplasmic	-2.02
Q96QR8	Transcriptional activator protein Pur-beta	-2.02
Q9Y3A5	Ribosome maturation protein SBDS	-2.01
O95782	AP-2 complex subunit alpha-1;	-1.99
Q16891	MICOS complex subunit MIC60	-1.94
Q08397	Lysyl oxidase homolog 1;	-1.78
P28066	Proteasome subunit alpha type-5;	-1.64
P08754	Guanine nucleotide-binding protein G(k) subunit alpha	-1.49
P22528	Cornifin-B	-1.45
P56134	ATP synthase subunit f, mitochondrial	-1.41
O43493	Trans-Golgi network integral membrane protein 2	-1.32
P08648	Integrin alpha-5;	-1.23
P05556	Integrin beta-1	-1.19
P12110	Collagen alpha-2(VI) chain	-1.01
Q07065	Cytoskeleton-associated protein 4	0.78
P30101	Protein disulfide-isomerase A3	0.97
Q13442	28 kDa heat- and acid-stable phosphoprotein	1.21
P08253	type IV collagenase;	1.30
Q13347	Eukaryotic translation initiation factor 3 subunit I;	1.33
Q14515	SPARC-like protein 1	1.53
Q01469	Fatty acid-binding protein	1.54
O00339	Matrilin-2	1.57
Q96CX2	BTB/POZ domain-containing protein KCTD12	1.58
Q02809	Procollagen-lysine,2-oxoglutarate 5-dioxygenase	1.63
O00391	Sulfhydryl oxidase 1;	1.70
P25815	Protein S100-P	1.77
P36952	Serpin B5;	1.88
P24821	Tenascin	1.91
Q16769	Glutaminyl-peptide cyclotransferase	1.93
P55058	Phospholipid transfer protein;	2.05
Q6UX71	Plexin domain-containing protein 2	2.08
O76061	Stanniocalcin-2	2.11
P52566	Rho GDP-dissociation inhibitor 2	2.13
P02771	Alpha-fetoprotein;	2.21
Q9NRN5	Olfactomedin-like protein 3	2.23
P39059	Collagen alpha-1(XV) chain;	2.27
P02746	Complement C1q subcomponent subunit B	2.29
P17900	Ganglioside GM2 activator	2.35
P06737	Glycogen phosphorylase, liver form;	2.38
Q9BY89	Uncharacterized protein KIAA167	2.44
Q9Y446	Plakophilin-3	2.44
Q4ZHG4	Fibronectin type III domain-containing protein 1	2.45
Q9Y6R7	Fc fragment of IgG binding protein	2.49
O94985	Calsyntenin	2.50
Q08380	Galectin-3-binding protein;	2.53
P10909	Clusterin	2.57
P07476	Involucrin	2.65
Q92817	Envoplakin	2.71
Q15113	Procollagen C-endopeptidase enhancer 1	2.73
P12236	ADP/ATP translocase 3	2.79
P43251	Biotinidase	2.81
P01861	Immunoglobulin heavy constant gamma 4	2.81
Q6P587	Acylpyruvase FAHD1	2.89
Q9UBC9	Small proline-rich protein 3	2.93
O95274	Ly6/PLAUR domain-containing protein	2.98
P19320	Vascular cell adhesion protein 1	3.01
P12830	Cadherin-1	3.05
Q99542	Matrix metalloproteinase-19	3.20
Q8IVL6	Prolyl 3-hydroxylase 3	3.35
P16144	Integrin beta-4;	3.36
Q9Y2B0	Protein canopy homolog 2	3.41
Q9Y240	C-type lectin domain family 11 member A;	3.47
Q16787	Laminin subunit alpha-3	3.50
Q02487	Desmocollin-2	3.69
P32926	Desmoglein-3	4.39
P47929	Lectin, galactoside-binding, soluble, 7B;	5.43

## Discussion

The mechanisms of development of FGR and PreE are likely multifactorial and poorly understood. The umbilical cord may have either a direct role in the pathophysiology or may have changes secondary to the underlying process. Elucidating changes that are similar and different in the two conditions may help to uncover the underlying mechanism of the disease process and identify novel targets for therapeutics.

Wharton’s Jelly is an important component of the umbilical cord and may contribute to the pathophysiology of many conditions including FGR and PreE. The normal development of the umbilical cord and Wharton’s Jelly has been previously characterized [25, 26]. The umbilical cord and Wharton’s Jelly increases in cross-sectional area until around 32 weeks’ gestation at which point it levels off for the remainder of the pregnancy. Umbilical cords that have a larger cross-sectional area are primarily driven by increased vessel area. Umbilical cords that are smaller than average are due to decreased Wharton’s Jelly area and are correlated to having a smaller placenta [26]. Previous studies have shown changes in the umbilical cord structure in FGR and PreE. The cross sectional area of the umbilical cord and Wharton’s Jelly has been shown to be significantly smaller in FGR [9, 27]. Additionally, vascular changes have been seen in patients with PreE with umbilical artery tunica media areas larger in the outer layer, inner layer, and lumen [12].

ECM is a complex tissue that includes many proteins which form basement membranes and interstitial structures. The role of the ECM is to provide biochemical and structural support for surrounding tissues. Additionally, the ECM is essential for tissue hydration, storage of growth factors, and is involved in the inflammatory process [28]. ECM is typically composed of proteoglycans, non-proteoglycan polysaccharides (such as hyaluronic acid), and other proteins including collagen and elastin. Changes to the composition and ratio of these proteins impacts the tissue both in terms of biochemical function and mechanical stiffness. Because of the essential role of the ECM, disruption can lead to many diseases [29]. Disruption of ECM formation and composition may either be a causative component of changes seen in umbilical cord structure in FGR and PreE or as a result of the underlying mechanism of this disease.

Interestingly, there are intrinsic differences in the inflammatory response of the umbilical cord vasculature with significant changes in immune response factors in the umbilical artery and vein [30]. During infection, there is an increase in IL-1β and IL-8 mRNA in the umbilical vein. Fibrocytes arise from monocyte precursors and have features of tissue remodeling fibroblasts and macrophages [31]. Fetal fibroblasts migrate into the Wharton’s Jelly via umbilical cord vasculature and there is significant decrease in the fibrocyte migration in FGR [30]. This decrease in fibrocytes in FGR may provide a mechanism for reduced stromal volume as fibroblasts are the major contributor to extracellular matrix production.

This study shows a significant decrease in umbilical cord area in FGR and FGR with PreE. This is driven by a decrease in the Wharton’s Jelly area similar to previous studies [27]. There were no significant differences in the area of the umbilical artery and vein. Of note, the FGR with PreE group did have a higher mean area of the tunica media inner layer, but this did not reach significance due to wide variability. The larger amount of variability in the FGR with PreE group may be driven by a separate mechanism underlying the disease process in PreE.

The data from this study shows significant changes across many cellular components and functions. The majority of proteins identified as downregulated in the FGR with PreE versus control group are involved with the extracellular matrix, RNA damage and repair, immune response, cell death pathways and cellular function. Proteins that were upregulated are involved with oxidative stress response, immune response, and cell adhesion. We also found a significant downregulation of pregnancy-zone protein in FGR with PreE versus controls.

When comparing FGR versus controls, the proteins identified fall into very different cellular function pathways. The majority of proteins that were downregulated in the FGR groups are involved with extracellular matrix formation including procollagen, collagen alpha-1 chain, and matrillin. Pregnancy-zone protein was also found to be downregulated in the FGR group as was alpha-fetoprotein. The physiologic role of pregnancy-zone protein and alpha-fetoprotein in the umbilical cord is unknown. The majority of proteins that were upregulated were involved with transcription factors, cell adhesion, ECM organization, and golgi transport.

The comparison of FGR with PreE versus isolated FGR revealed eighty significantly changed proteins. Inflammatory response proteins had mixed expression changes across the different groups. Many of the downregulated proteins are involved with cell energy pathways, exocytosis, and metabolic pathways. Upregulated pathways include vasculature remodeling, angiogenesis, and ECM composition and development. Previous studies have shown an upregulation of pro-inflammatory substances and mixed changes in angiogenic and anti-angiogenic factors in isolated PreE [32]. Here we see a similar mixture of up and down-regulation of angiogenic factors likely displaying the complex nature of the PreE process.

This study is novel as it is the first to use proteomics to specifically look at the proteomic changes in the Wharton’s Jelly, which has been understudied compared to other tissue such as maternal and fetal blood and placenta. The results of this study show the significant changes in the proteins that serve as significant components of the ECM and likely contribute to the significant reductions in umbilical cord cross-sectional area and Wharton’s Jelly. A strength of this study is the novel use of five protein search and inference engines for identification of proteins from the mass spectrometry data as well as using both spectral count and peak intensity methods. By using the overlap data of commonly identified proteins that were significantly changed, this increases the confidence that this is a true change.

A recent meta-analysis of proteomic biomarkers for PreE identified many differentially expressed proteins in multiple biological samples including maternal blood, placenta, umbilical cord blood and urine [14]. Our study demonstrated multiple significant changes in proteins highlighted in this meta-analysis. Fibrinogen alpha chain was found to be increased in the PreE+FGR group compared the FGR. This protein was also found to be increased in blood and urine samples in the meta-analysis. Similar patterns were seen for clusterin. We found that pregnancy -zone protein was significantly reduced in our FGR versus control group. The meta-analysis showed a significant decrease in this protein in blood, but an increase in plasma and urine in women with PreE. The meta-analysis study highlights the complex differential expression of proteins in different tissue samples from women with PreE. Our study adds to the knowledge base by including an additional tissue type further demonstrating the complexity of protein expression between both FGR and FGR+PreE.

Limitations to the study include a small number of patients for each group. Increasing the numbers may help to decrease the variance in the histological data and proteomic data. The route of delivery was not proportional in each of the groups, but route of delivery is unlikely to contribute to change in protein expression or structure. Another limitation is that there was no isolated PreE group for comparison. It is interesting to see the proteomic changes with those with FGR that did develop PreE compared to isolated FGR as they are likely distinct process, but it does not allow for comparison of isolated PreE versus controls which all the other studies have previously compared. Additionally, the proteomics results can serve as a screening tool where proteins identified as significantly changed will need to be confirmed by traditional validation methods such as western blotting.

Identification of changes to the proteomic profiles and mechanism of umbilical cord morphology changes may identify novel targets as potential mechanisms for FGR and PreE. We demonstrated that there is a significant reduction in umbilical cord area and Wharton’s Jelly area in FGR, similar to previous studies. Proteomic analysis showed changes in extracellular matrix, cellular process, inflammatory pathway, and angiogenesis proteins across the group comparisons. These results display the complex nature of the changes in the umbilical cord and additional research into these changes may help to identify the mechanisms behind FGR and PreE. Further work in validating these proteomic differences may enable identification of novel molecules or pathways for therapeutic targets for these conditions.

## Supporting information

S1 Fig Artery average tunica media outer layer area. There were no significant differences (p = 0.466).
(TIF)

S2 Fig Artery average tunica media inner layer area. There were no significant differences (p = 0.754).
(TIF)

S3 Fig Umbilical cord vein area. There were no significant differences (p = 0.794).
(TIF)

S4 Fig Overlap of significantly changed protein ID’s from mass spectrometry search and inference engines for spectral count of FGR versus controls. Legend (CF: comet fido; CE: comet epifany; XE: X!Tandem epifany; MF: MSGF fido; ME: MSGF epiphany).
(TIF)

S5 Fig Overlap of significantly changed protein ID’s from mass spectrometry search and inference engines for peak intensity of FGR+PreE versus controls. Legend (CF: comet fido; CE: comet epifany; XE: X!Tandem epifany; MF: MSGF fido; ME: MSGF epiphany).
(TIF)

S6 Fig Overlap of significantly changed protein ID’s from mass spectrometry search and inference engines for spectral count of FGR+PreE versus controls. Legend (CF: comet fido; CE: comet epifany; XE: X!Tandem epifany; MF: MSGF fido; ME: MSGF epiphany).
(TIF)

S7 Fig Overlap of significantly changed protein ID’s from mass spectrometry search and inference engines for peak intensity of FGR+PreE versus FGR. Legend (CF: comet fido; CE: comet epifany; XE: X!Tandem epifany; MF: MSGF fido; ME: MSGF epiphany).
(TIF)

S8 Fig Overlap of significantly changed protein ID’s from mass spectrometry search and inference engines for spectral count of FGR+PreE versus FGR. Legend (CF: comet fido; CE: comet epifany; XE: X!Tandem epifany; MF: MSGF fido; ME: MSGF epiphany).
(TIF)

S1 Table Intersection of significantly changed protein expression for FGR versus controls peak intensity. (DOCX)

S2 Table Intersection of significantly changed protein expression for FGR+PreE versus controls spectral count. (DOCX)

## References

[1] Fetal Growth Restriction: ACOG Practice Bulletin Summary, Number 227. Obstetrics & Gynecology 2021; 137. 10.1097/AOG.0000000000004252 33481523

[2] Barker DJ,. Adult consequences of fetal growth restriction. Clin.Obstet.Gynecol. 2006; 49:270–283. doi: 10.1097/00003081-200606000-00009 16721106

[3] Pallotto EK, Kilbride HW,. Perinatal outcome and later implications of intrauterine growth restriction. Clin.Obstet.Gynecol. 2006; 49:257–269. doi: 10.1097/00003081-200606000-00008 16721105

[4] Salafia CM, Minior VK, Pezzullo JC, Popek EJ, Rosenkrantz TS, Vintzileos AM,. Intrauterine growth restriction in infants of less than thirty-two weeks’ gestation: associated placental pathologic features. Am.J.Obstet.Gynecol. 1995; 173:1049–1057. doi: 10.1016/0002-9378(95)91325-4 7485292

[5] Backes CH, Markham K, Moorehead P, Cordero L, Nankervis CA, Giannone PJ,. Maternal Preeclampsia and Neonatal Outcomes. Journal of Pregnancy 2011; 2011:7. doi: 10.1155/2011/214365 21547086 PMC3087144

[6] Dutman AC, Nikkels PGJ,. Umbilical Hypercoiling in 2nd- and 3rd-Trimester Intrauterine Fetal Death. Pediatr.Dev.Pathol. 2015; 18:10–16. doi: 10.2350/13-10-1390-OA.1 25360762

[7] Sobolewski K, Bankowski E, Chyczewski L, Jaworski S,. Collagen and glycosaminoglycans of Wharton’s jelly. Biol.Neonate 1997; 71:11–21. doi: 10.1159/000244392 8996653

[8] Bańkowski E, Sobolewski K, Romanowicz L, Chyczewski L, Jaworski S,. Collagen and glycosaminoglycans of Wharton’s jelly and their alterations in EPH-gestosis. European Journal of Obstetrics and Gynecology 1996; 66:109–117. doi: 10.1016/0301-2115(96)02390-1 8735730

[9] Peyter A.-C.|Delhaes F.|Baud D.|Vial Y.|Diaceri G.|Menétrey, et al.,. Intrauterine growth restriction is associated with structural alterations in human umbilical cord and decreased nitric oxide-induced relaxation of umbilical vein. Placenta 2014; 35:891–899. doi: 10.1016/j.placenta.2014.08.090 25249153

[10] Bruch JF, Sibony O, Benali K, Challier JC, Blot P, Nessmann C,. Computerized microscope morphometry of umbilical vessels from pregnancies with intrauterine growth retardation and abnormal umbilical artery doppler. Human Pathology 1997; 28:1139–1145. doi: 10.1016/s0046-8177(97)90251-3 9343320

[11] Raio L, Ghezzi F, Di Naro E, Duwe DG, Cromi A, Schneider H,. Umbilical cord morphologic characteristics and umbilical artery Doppler parameters in intrauterine growth-restricted fetuses. J.Ultrasound Med. 2003; 22:1341–1347. doi: 10.7863/jum.2003.22.12.1341 14682422

[12] blanco m, Vega H, Giuliano R, Grana D, Azzato F, Lerman J, et al. Histomorphometry of Umbilical Cord Blood Vessels in Preeclampsia. The Journal of Clinical Hypertension 2011; 13:30–34. doi: 10.1111/j.1751-7176.2010.00384.x 21214719 PMC8673216

[13] Law KP, Han TL, Tong C, Baker PN,. Mass spectrometry-based proteomics for pre-eclampsia and preterm birth. Int.J.Mol.Sci. 2015; 16:10952–10985. doi: 10.3390/ijms160510952 26006232 PMC4463685

[14] Nguyen TPH, Patrick CJ, Parry LJ, Familari M,. Using proteomics to advance the search for potential biomarkers for preeclampsia: A systematic review and meta-analysis. PLoS One 2019; 14:e0214671. doi: 10.1371/journal.pone.0214671 30951540 PMC6450632

[15] Dahabiyeh LA,. The discovery of protein biomarkers in pre-eclampsia: the promising role of mass spectrometry. Biomarkers 2018; 23:609–621. doi: 10.1080/1354750X.2018.1474257 29737875

[16] Committee Opinion No 700: Methods for Estimating the Due Date. Obstet.Gynecol. 2017; 129:e150–e154. doi: 10.1097/AOG.0000000000002046 28426621

[17] Kito K, Ito T,. Mass spectrometry-based approaches toward absolute quantitative proteomics. Curr.Genomics 2008; 9:263–274. doi: 10.2174/138920208784533647 19452043 PMC2682933

[18] Bondarenko PV, Chelius D, Shaler TA,. Identification and relative quantitation of protein mixtures by enzymatic digestion followed by capillary reversed-phase liquid chromatography-tandem mass spectrometry. Anal.Chem. 2002; 74:4741–4749. doi: 10.1021/ac0256991 12349978

[19] Eng JK, Jahan TA, Hoopmann MR,. Comet: an open-source MS/MS sequence database search tool. Proteomics 2013; 13:22–24. doi: 10.1002/pmic.201200439 23148064

[20] Pfeuffer J, Sachsenberg T, Dijkstra TMH, Serang O, Reinert K, Kohlbacher O,. EPIFANY: A Method for Efficient High-Confidence Protein Inference. J.Proteome Res. 2020; 19:1060–1072. doi: 10.1021/acs.jproteome.9b00566 31975601 PMC7583457

[21] Kim S, Pevzner PA,. MS-GF+ makes progress towards a universal database search tool for proteomics. Nat.Commun. 2014; 5:5277. doi: 10.1038/ncomms6277 25358478 PMC5036525

[22] Craig R, Beavis RC,. TANDEM: matching proteins with tandem mass spectra. Bioinformatics 2004; 20:1466–1467. doi: 10.1093/bioinformatics/bth092 14976030

[23] Lun AT, Chen Y, Smyth GK,. It’s DE-licious: A Recipe for Differential Expression Analyses of RNA-seq Experiments Using Quasi-Likelihood Methods in edgeR. Methods Mol.Biol. 2016; 1418:391–416. doi: 10.1007/978-1-4939-3578-9_19 27008025

[24] Gardner ML, Freitas MA,. Multiple Imputation Approaches Applied to the Missing Value Problem in Bottom-up Proteomics. bioRxiv 2020:2020.06.29.178335. 10.3390/ijms22179650 PMC8431783 34502557

[25] Barbieri C, Cecatti JG, Surita FG, Costa ML, Marussi EF, Costa JV,. Area of Wharton’s jelly as an estimate of the thickness of the umbilical cord and its relationship with estimated fetal weight. Reprod.Health. 2011; 8:32–32. doi: 10.1186/1742-4755-8-32 22054163 PMC3219549

[26] Proctor L.K.|Fitzgerald B.|Whittle W.L.|Mokhtari N.|Lee E.|Machin, et al.,. Umbilical cord diameter percentile curves and their correlation to birth weight and placental pathology. Placenta 2012; 34:62–66. doi: 10.1016/j.placenta.2012.10.015 23174148

[27] Sharony, Reuven Keltz, Eran Biron-Shental, Tal Kidron, Debora,. Morphometric characteristics of the umbilical cord and vessels in fetal growth restriction and pre-eclampsia. Early Human Development 2015; 92:57–62. doi: 10.1016/j.earlhumdev.2015.11.006 26678004

[28] LEVER R, SMAILBEGOVIC A, PAGE C,. Role of glycosaminoglycans in in ammation. inflammopharmacology 2001; 9, No 1,2:165–169.

[29] Byron Adam, Humphries Jonathan, Humphries Martin,. Defining the extracellular matrix using proteomics. Int.J.Exp.Pathol. 2013; 94:75–92. doi: 10.1111/iep.12011 23419153 PMC3607136

[30] Kim J, Romero R, Tarca AL, LaJeunesse C, Han YM, Kim MJ, et al,. Gene expression profiling demonstrates a novel role for foetal fibrocytes and the umbilical vessels in human fetoplacental development. Journal of Cellular and Molecular Medicine 2008; 12:1317–1330. doi: 10.1111/j.1582-4934.2008.00284.x 18298660 PMC2837362

[31] Reilkoff RA, Bucala R, Herzog EL,. Fibrocytes: emerging effector cells in chronic inflammation. Nature reviews.Immunology 2011; 11:427–435. doi: 10.1038/nri2990 21597472 PMC3599774

[32] Lau SY, Guild S, Barrett CJ, Chen Q, McCowan L, Jordan V, et al,. Tumor Necrosis Factor‐Alpha, Interleukin‐6, and Interleukin‐10 Levels are Altered in Preeclampsia: A Systematic Review and Meta‐Analysis. American Journal of Reproductive Immunology 2013; 70:412–427. doi: 10.1111/aji.12138 23790133

