# Supplementary figures and images for: Proteomic analysis of the umbilical cord in fetal growth restriction and preeclampsia
Source: PLoS One. 2022 Feb 25;17(2):e0262041. doi: 10.1371/journal.pone.0262041 (PMC8880394; doi:10.1371/journal.pone.0262041)

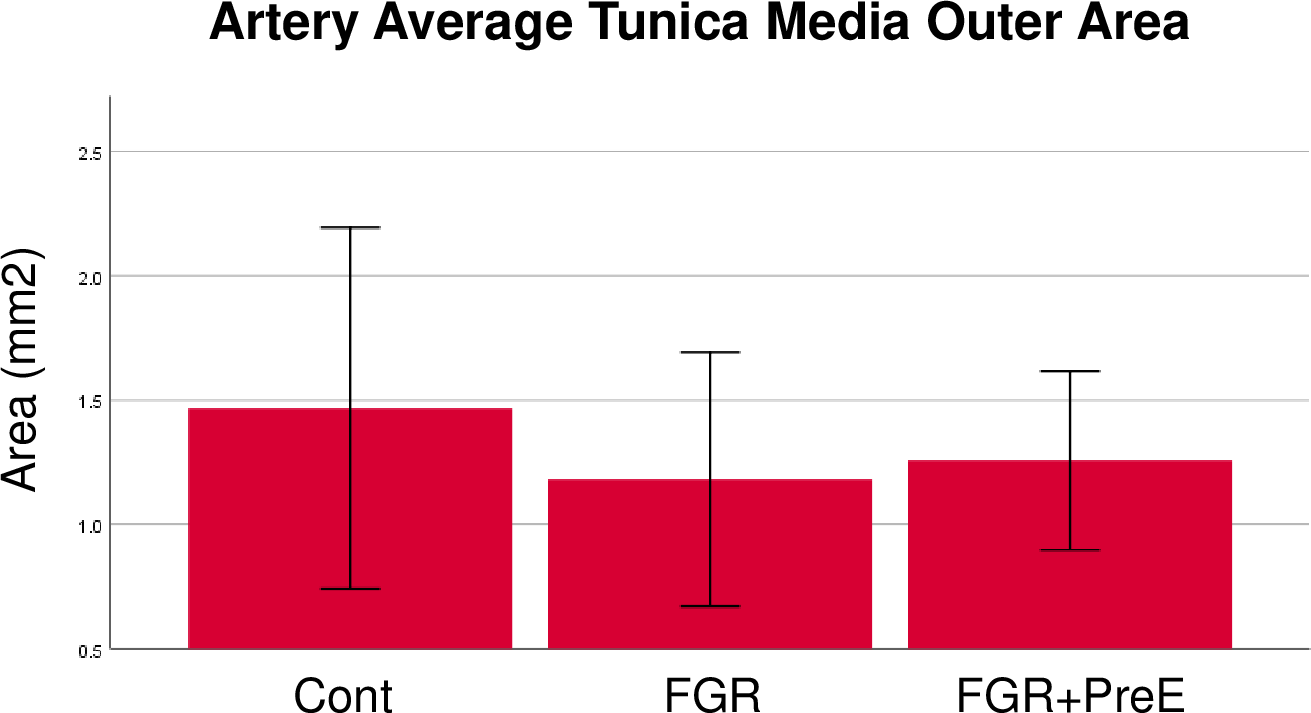

Supplement: S1 Fig — There were no significant differences (p = 0.466). (TIF) [file pone.0262041.s001.tif]

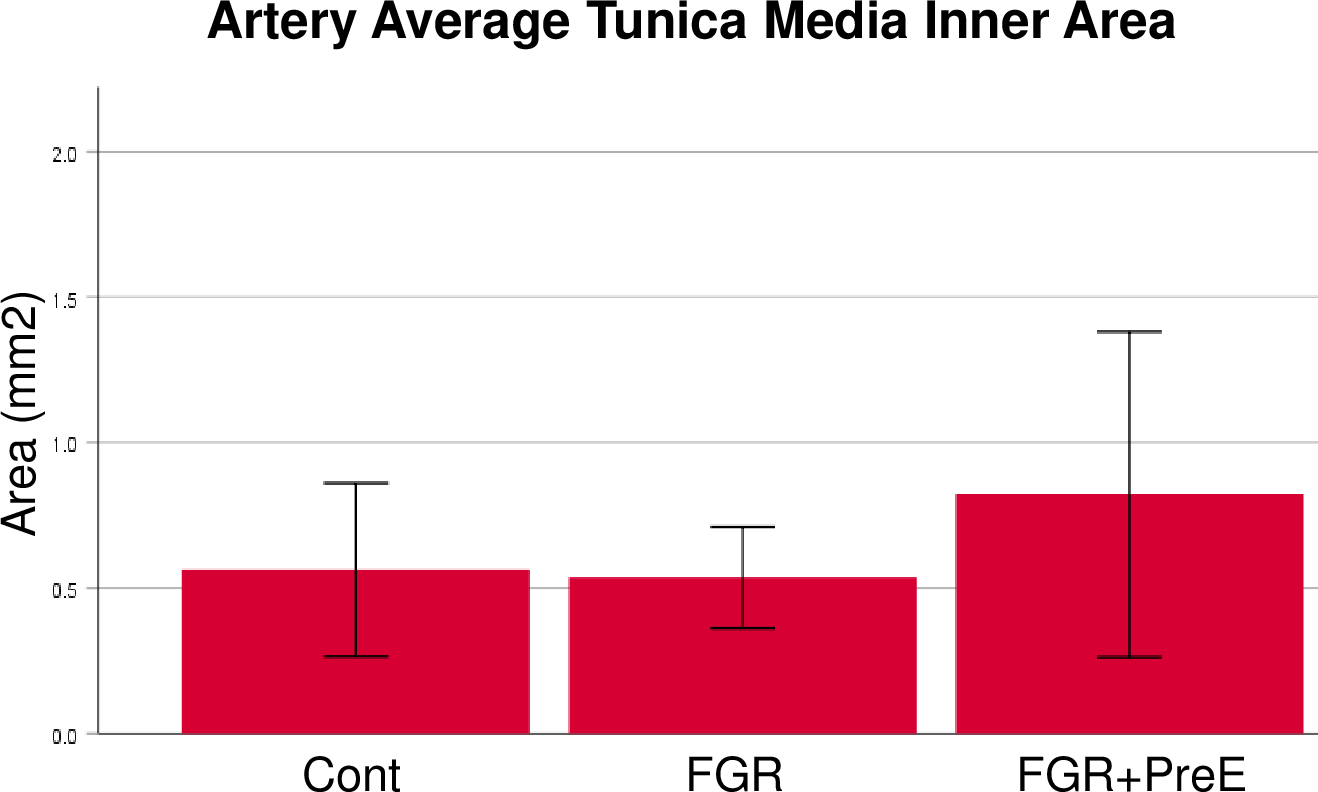

Supplement: S2 Fig — There were no significant differences (p = 0.754). (TIF) [file pone.0262041.s002.tif]

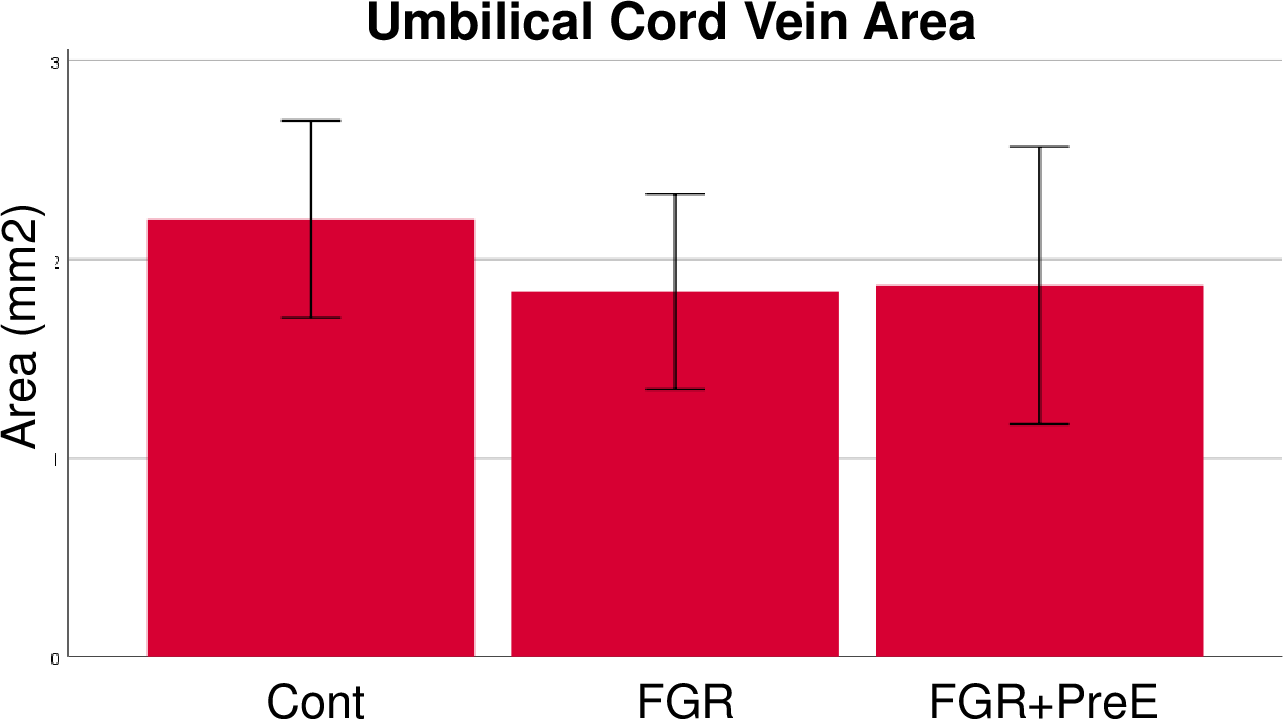

Supplement: S3 Fig — There were no significant differences (p = 0.794). (TIF) [file pone.0262041.s003.tif]

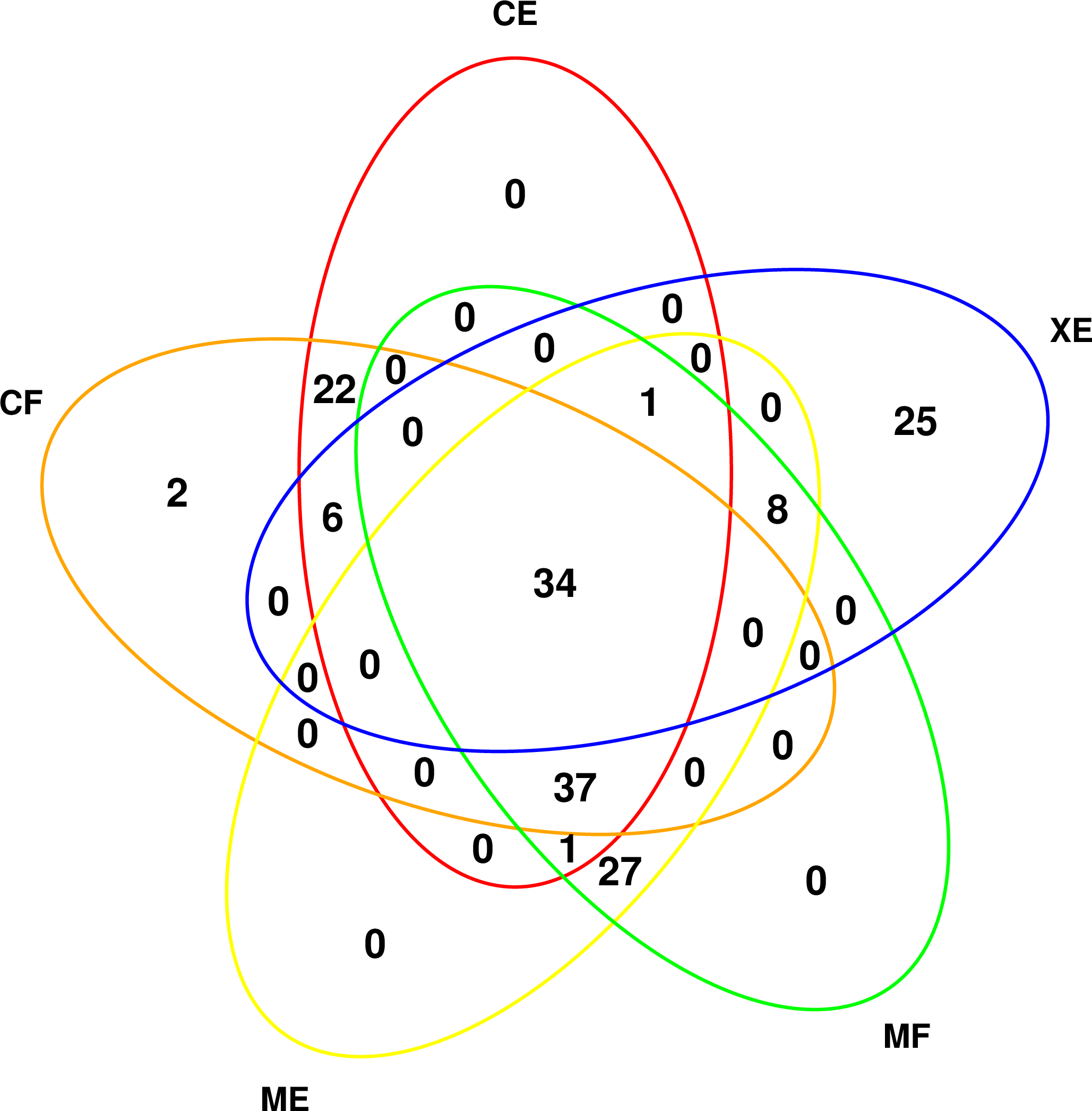

Supplement: S4 Fig — Legend (CF: comet fido; CE: comet epifany; XE: X!Tandem epifany; MF: MSGF fido; ME: MSGF epiphany). (TIF) [file pone.0262041.s004.tif]

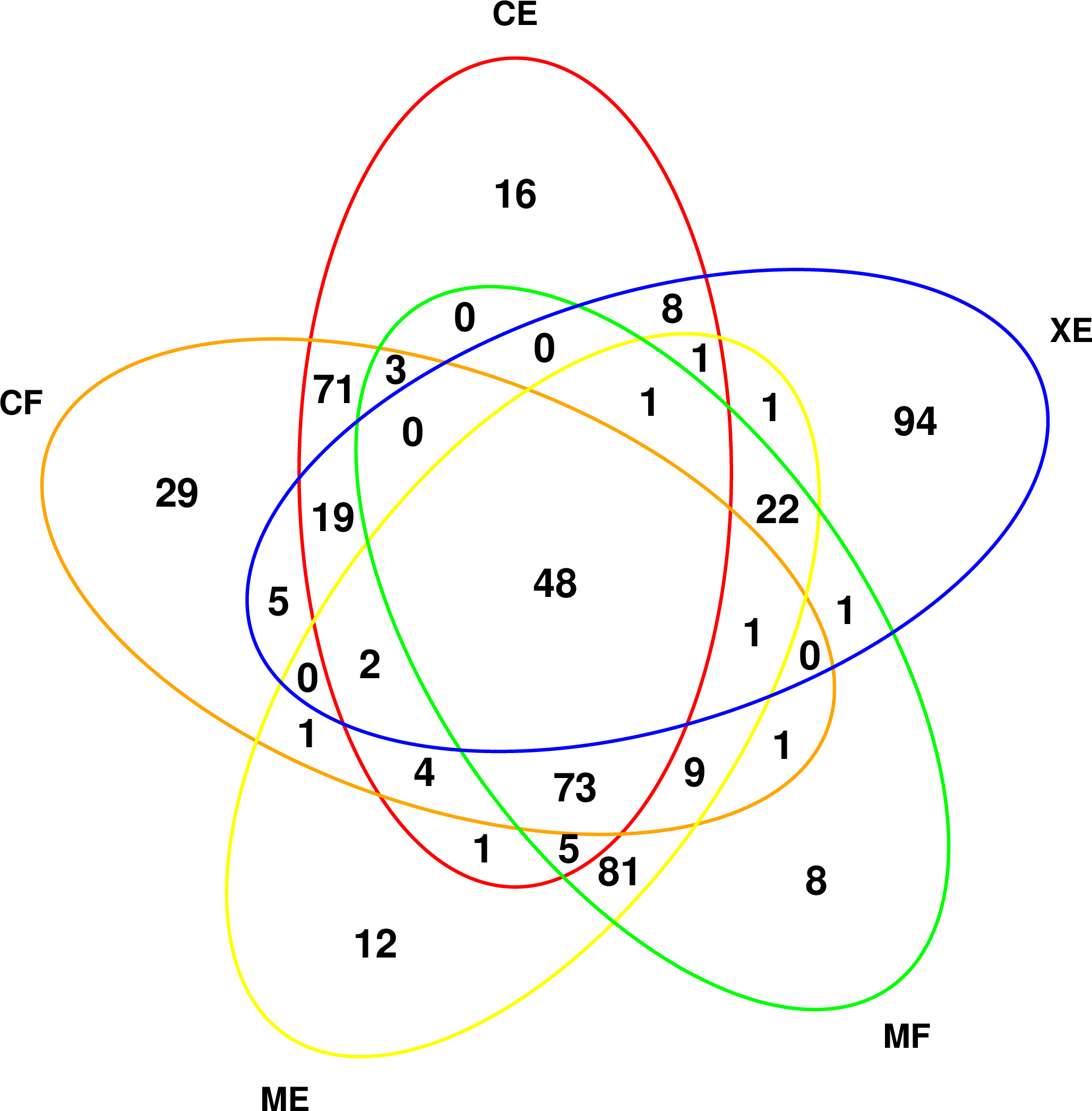

Supplement: S5 Fig — Legend (CF: comet fido; CE: comet epifany; XE: X!Tandem epifany; MF: MSGF fido; ME: MSGF epiphany). (TIF) [file pone.0262041.s005.tif]

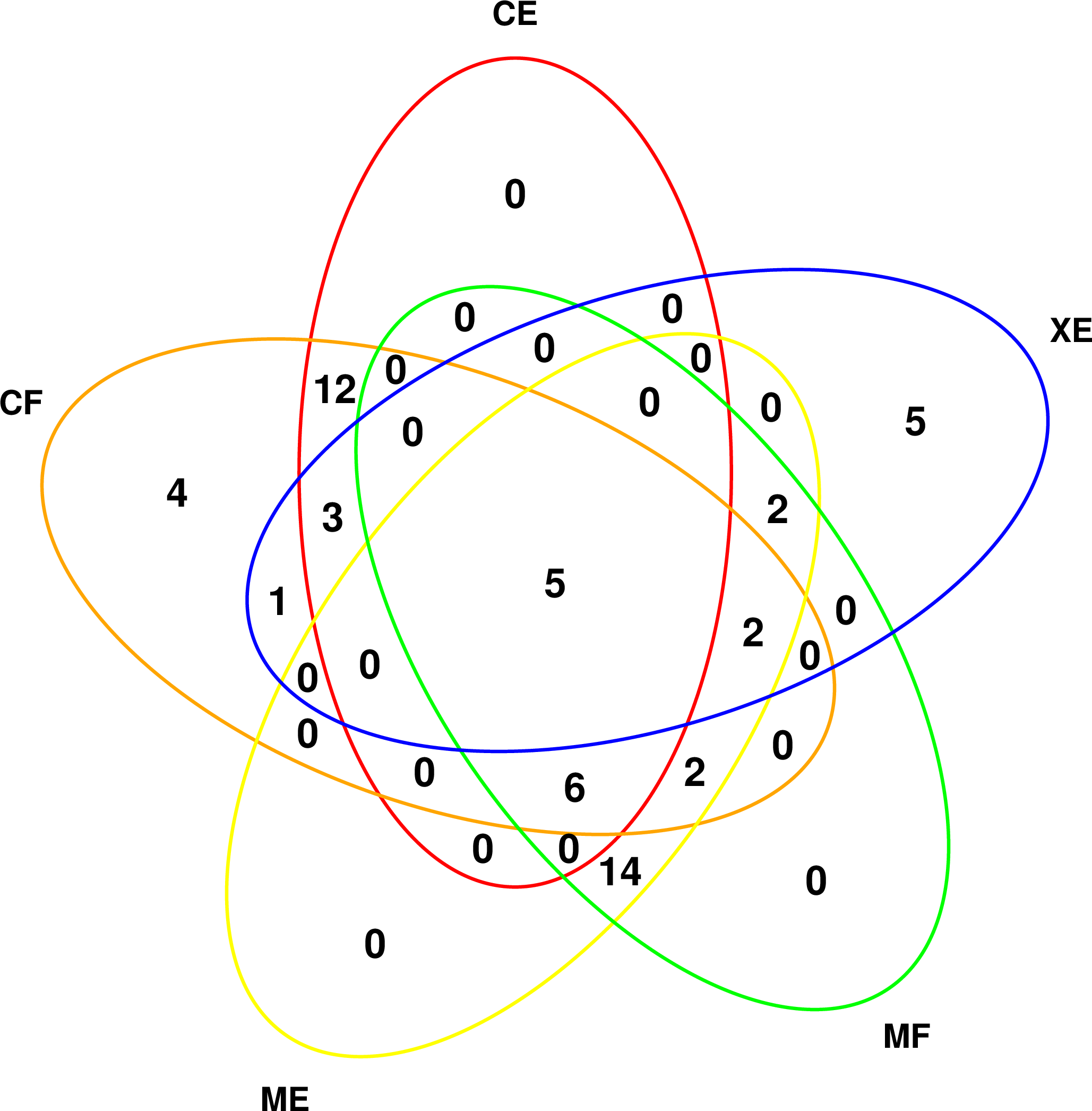

Supplement: S6 Fig — Legend (CF: comet fido; CE: comet epifany; XE: X!Tandem epifany; MF: MSGF fido; ME: MSGF epiphany). (TIF) [file pone.0262041.s006.tif]

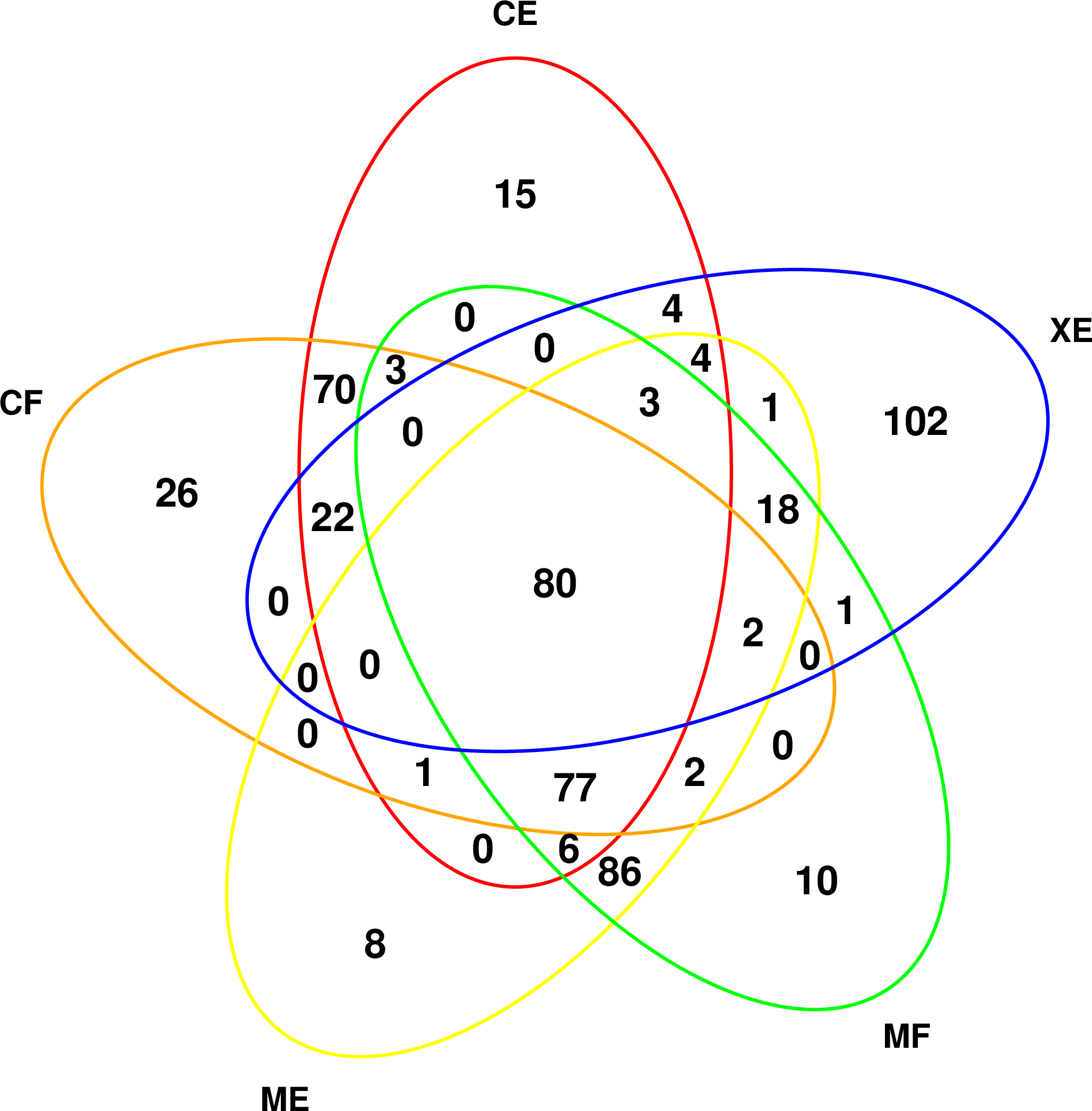

Supplement: S7 Fig — Legend (CF: comet fido; CE: comet epifany; XE: X!Tandem epifany; MF: MSGF fido; ME: MSGF epiphany). (TIF) [file pone.0262041.s007.tif]

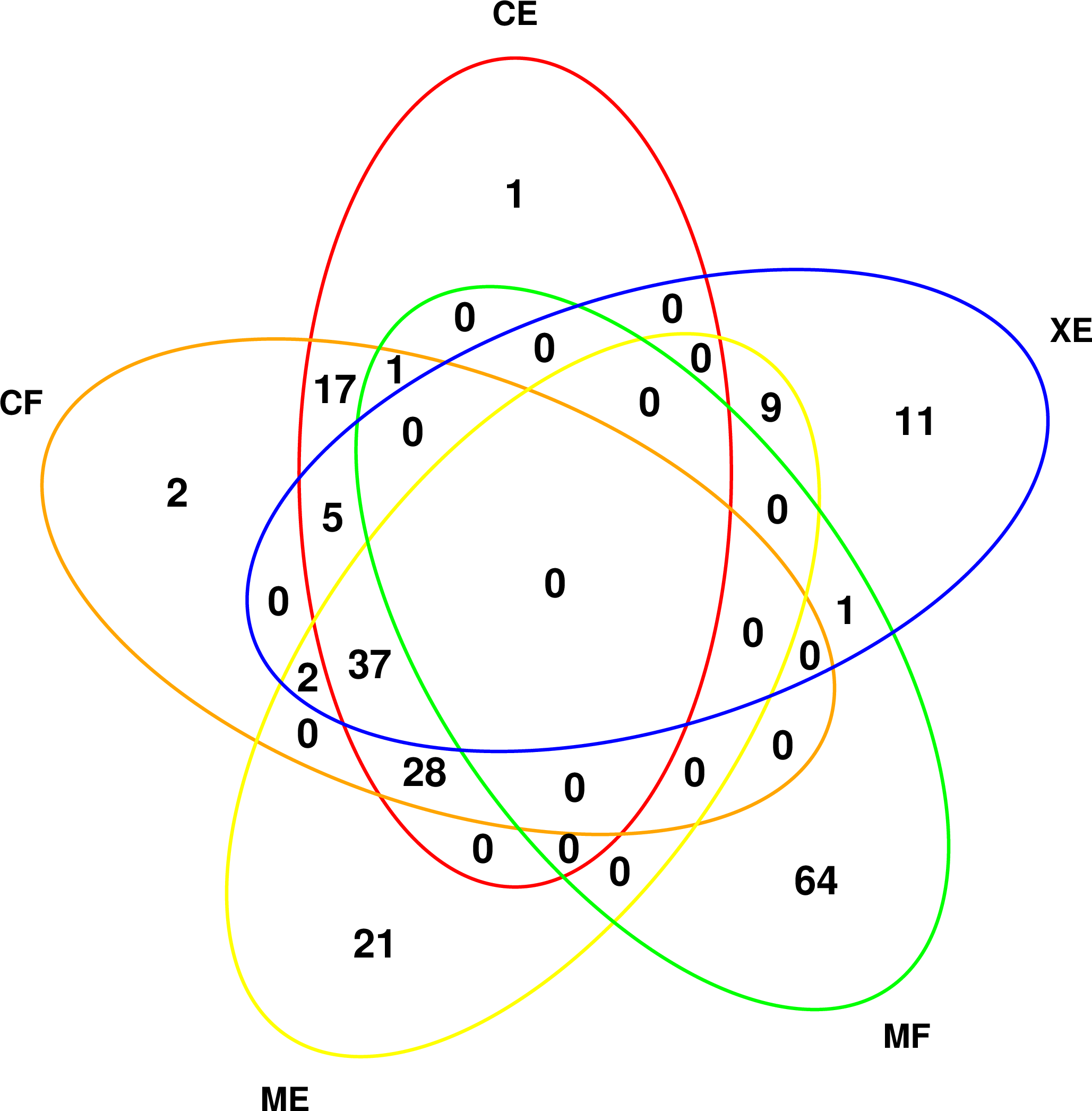

Supplement: S8 Fig — Legend (CF: comet fido; CE: comet epifany; XE: X!Tandem epifany; MF: MSGF fido; ME: MSGF epiphany). (TIF) [file pone.0262041.s008.tif]
