# Supplementary material for: Proteomic analysis of the umbilical cord in fetal growth restriction and preeclampsia
Source: PLoS One. 2022 Feb 25;17(2):e0262041. doi: 10.1371/journal.pone.0262041 (PMC8880394; doi:10.1371/journal.pone.0262041)
Supplement: S1 Table — (DOCX) [file pone.0262041.s009.docx]

**S1 Table. Intersection of significantly changed protein expression for FGR versus controls peak intensity.**

| **UniProt ID** | **Protein** | **Log Fold Change** |
| --- | --- | --- |
| Q8IVL6 | Prolyl 3-hydroxylase 3 | -4.09 |
| P01861 | Immunoglobulin heavy constant gamma 4 | -3.60 |
| Q9Y2B0 | Protein canopy homolog 2 | -3.19 |
| Q15113 | Procollagen C-endopeptidase enhancer 1 | -3.13 |
| Q96IJ6 | Mannose-1-phosphate guanyltransferase alpha | -3.08 |
| P17900 | Ganglioside GM2 activator | -2.92 |
| P49755 | Transmembrane emp24 domain-containing protein 10 | -2.40 |
| Q02809 | Procollagen-lysine,2-oxoglutarate 5-dioxygenase 1 | -2.36 |
| P31947 | 14-3-3 protein sigma | -2.05 |
| P39059 | Collagen alpha-1(XV) chain | -2.03 |
| Q13442 | 28 kDa heat- and acid-stable phosphoprotein | -1.89 |
| P24821 | Tenascin | -1.56 |
| O00339 | Matrilin-2 | -1.51 |
| P40616 | ADP-ribosylation factor-like protein 1 | -1.00 |
| P08648 | Integrin alpha-5 | 1.25 |
| Q9BWD1 | Acetyl-CoA acetyltransferase, cytosolic | 1.40 |
| O14579 | Coatomer subunit epsilon | 1.60 |
| O15230 | Laminin subunit alpha-5 | 1.82 |
| P08574 | Cytochrome c1, heme protein, mitochondrial | 2.36 |
| O14672 | ADAM 10 | 2.57 |
| Q9GZM7 | Tubulointerstitial nephritis antigen-like | 2.69 |
| P35998 | 26S proteasome regulatory subunit 7 | 2.71 |
| Q96IU4 | Protein ABHD14B | 3.23 |
| Q9UGI8 | Testin | 3.39 |
| O60763 | General vesicular transport factor p115 | 3.46 |
| Q9UJZ1 | Stomatin-like protein 2, mitochondrial | 3.48 |
| Q00534 | Cyclin-dependent kinase 6 | 3.88 |
| Q9UNS2 | COP9 signalosome complex subunit 3 | 4.49 |
