# Supplementary material for: Proteomic analysis of the umbilical cord in fetal growth restriction and preeclampsia
Source: PLoS One. 2022 Feb 25;17(2):e0262041. doi: 10.1371/journal.pone.0262041 (PMC8880394; doi:10.1371/journal.pone.0262041)
Supplement: S2 Table — (DOCX) [file pone.0262041.s010.docx]

**S2 Table. Intersection of significantly changed protein expression for FGR+PreE versus controls spectral count.**

| **UniProt ID** | **Protein** | **Log Fold Change** |
| --- | --- | --- |
| P20742 | Pregnancy zone protein | -2.94 |
| P54886 | Delta-1-pyrroline-5-carboxylate synthase | -1.32 |
| P06737 | Glycogen phosphorylase, liver form | 1.17 |
| Q9UBC9 | Small proline-rich protein 3 | 2.71 |
| P53634 | Dipeptidyl peptidase 1 | 3.78 |
